# Pre-hospital care for children: a descriptive study from Central Norway

**DOI:** 10.1186/s13049-024-01279-x

**Published:** 2024-11-04

**Authors:** Martine Myhre, Lars Eide Næss, Eirik Skogvoll, Helge Haugland

**Affiliations:** 1 Faculty of Medicine and Health Science, Norwegian University of Science and Technology (NTNU), Trondheim, Norway; 2 Department of Emergency Medicine and Pre-Hospital Services, St. Olav’s University Hospital, Trondheim, Norway; 3 Department of Research and Development, The Norwegian Air Ambulance Foundation, Oslo, Norway; 4 Department of Circulation and Medical Imaging, Faculty of Medicine and Health Science, Norwegian University of Science and Technology (NTNU), Trondheim, Norway; 5 Department of Anesthesiology and Intensive Care Medicine, St. Olav’s University Hospital, Trondheim, Norway

**Keywords:** Pediatric emergency medicine, Epidemiology of pediatric emergencies, Emergency medical service, Pediatric emergency treatment, Physician-staffed emergency medical service, Helicopter emergency medical service

## Abstract

**Background:**

Pre-hospital incidents involving pediatric and neonatal patients are infrequent, and clinical characteristics and care for these patients differ from the adult population. Lack of knowledge, guidelines, and experience can make pre-hospital pediatric care challenging, and there is limited research on the epidemiology and best practice of care for this population. We examined the pre-hospital pediatric population in the county of Sør-Trøndelag, Norway, to improve our understanding of this population in our region.

**Methods:**

We conducted a retrospective observational cohort study of emergency incidents involving children under twelve years of age with dispatch of Emergency Medical Services (EMS) in Sør-Trøndelag between 2018 and 2022. Incidents and patient characteristics were extracted from the Emergency Medical Communication Center (EMCC) database. In addition, data on patient characteristics and interventions for more serious incidents seen by the Helicopter Emergency Medical Service (HEMS) were included from the database LABAS. We provided descriptive statistics and estimated population incidences using Poisson regression.

**Results:**

The catchment area of EMCC Sør-Trøndelag has a population of approximately 43,000 children under the age of twelve years. During the five-year study period, there were 7005 emergency calls concerning this patient population, representing 6% of all emergency calls (total no. 108,717). Of these, 3500 (50%) resulted in the dispatch of an ambulance and/or HEMS, yielding an annual incidence of EMS dispatches of 17 per 1000 children. The three most common primary medical problems were respiratory distress, altered consciousness, and trauma. Among the 309 HEMS patients, 131 (42%) received advanced interventions from the HEMS physician. Assisted ventilation was the most frequent intervention.

**Conclusions:**

Pediatric and neonatal patients make up a small proportion of pre-hospital patient dispatches in Sør-Trøndelag. Consequently, each EMS provider infrequently encounters children in the pre-hospital environment, resulting in less experience with pediatric advanced medical interventions. This study identifies some clinical characteristics and interventions regarding pediatric and neonatal patients that have been pointed out as focus areas for pediatric pre-hospital research.

**Supplementary Information:**

The online version contains supplementary material available at 10.1186/s13049-024-01279-x.

## Background

Children represent a small portion of the total emergency medical service (EMS) patients. The reported proportion of EMS contacts regarding pediatric and neonate patients varies between 4% in Finland, 5% in Canada, and 7% in Denmark [1–3]. Moreover, children constitute a diverse patient group, with each age group requiring different approaches. Neonates and young children have different medical problems and anatomical characteristics compared to older children. EMS providers must have the appropriate training, equipment, and protocols to treat children in different age groups.

The Pediatric Emergency Care Applied Research Network (PECARN) has developed a priority list identifying high-priority topics for pediatric EMS research. The top ten clinical priorities include airway management, respiratory distress, trauma, asthma, head trauma, shock, pain, seizures, respiratory arrest, and C-spine immobilization [4]. PECARN emphasizes the importance of research on pediatric pre-hospital care for several reasons. Firstly, the needs of children treated in the pre-hospital setting are different from those of adults. Children cannot be treated simply as smaller adults as they differ with respect to assessment, pathophysiology, equipment, and drug dosing. Secondly, research conducted on pediatric pre-hospital care will enable us to improve education, training, and care tailored to children, rather than relying on research on adults. By offering a pediatric-specific EMS research agenda, PECARN provides guidance for future pediatric pre-hospital research [4].

Hansen et al. conducted a Delphi survey in 2012 to identify knowledge gaps in pediatric pre-hospital emergency care [5]. The participants included paramedics, nurses, physicians, and other pre-hospital providers and identified the knowledge gaps that lead to patient safety concerns. Three of the most common knowledge gaps were lack of experience with pediatric airway management, lack of proficiency in pediatric skills, and lack of experience with pediatric equipment [5].

EMS staff repeatedly report significant challenges and heightened anxiety when attending to pediatric patients [5–8]. These may lead to patient safety hazards, e.g., problems with drug calculation and proper procedural performance [7, 9–12]. Cognitive aids, education, and evaluation of the most frequent situations have been proposed to address these challenges [1, 8, 13]. There is a lack of knowledge concerning the epidemiology of pediatric patients encountered by these services [14–16]. Knowledge of diagnosis, interventions, and outcomes can help identify which topics to address in education and training.

Children constitute a small and diverse patient group. In order to provide appropriate pre-hospital care, it is essential to understand the characteristics and needs of pediatric patients cared for by emergency medical service (EMS). This study aimed to describe the pediatric population in Central Norway. A secondary aim was to describe in more detail the characteristics of the children in contact with HEMS.

## Methods

This was a retrospective, observational cohort study of all emergency calls to the emergency medical communication center (EMCC) that led to the dispatch of ambulance and/or HEMS for children under twelve years of age in the county of Sør-Trøndelag in Central Norway. We used the Strengthening the Reporting of Observational Studies in Epidemiology (STROBE) guidelines as the underlying framework for this paper [17].

### Study setting

Norway has a publicly funded healthcare system, and EMS is free of charge for all citizens. These services include EMCCs, ambulances, and helicopter emergency medical services. Patients transported by EMS are generally admitted to either an emergency department in a hospital or to the general practitioners in local practices and out-of-hours primary health care services managed by the municipalities [18]. A similar publicly funded structure is established in all Scandinavian countries [19].

The EMCCs handle all medical emergency calls and coordinate the pre-hospital resources in their respective regions. Trained paramedics and nurses at the EMCCs use the “Norwegian Index of Medical Emergencies”, an algorithm-based digital decision support tool to assess the severity of incidents [20]. The EMCC operator decides the primary medical problem at the time of the emergency call based on the information provided by the caller.

Most pre-hospital incidents are handled by ambulances, staffed by paramedics or nurses. In need of more specialized competence, in remote areas or when reducing time to definitive care is possible with air transport, HEMS might be dispatched. This is a 24/7/365 service, staffed by a pilot, a specially trained flight paramedic or nurse, and a board-certified anesthesiologist (HEMS physician) [21]. Regional dispatch guidelines indicate when HEMS should be used (Supplementary file 1). If the patient is located near the HEMS base or the weather conditions do not permit flying, a rapid response car is used by the HEMS crew. The rapid response car increases the availability of advanced pre-hospital life support in the HEMS region [22]. However, it is dependent on an ambulance for patient transportation.

**Fig. 1:**
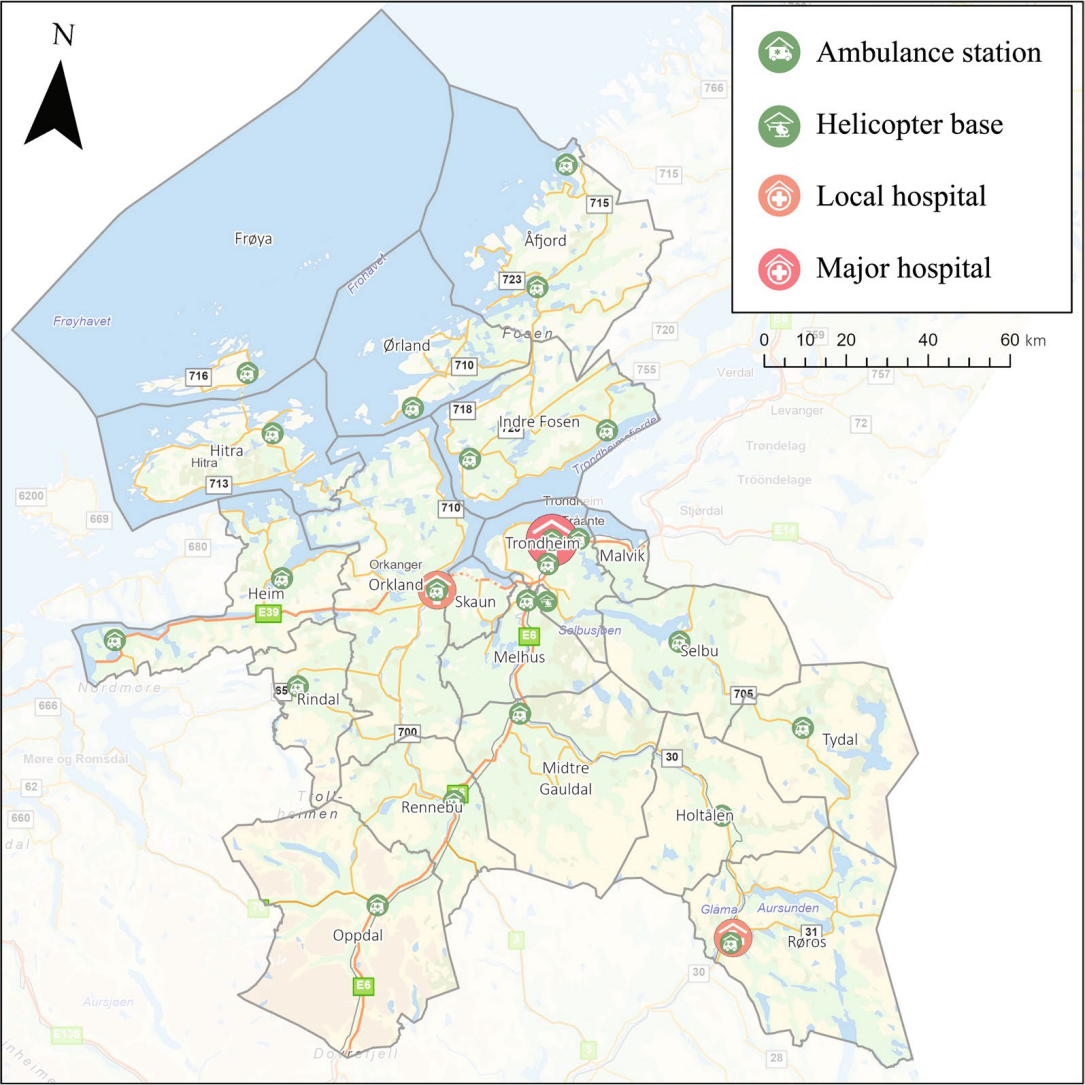
Ambulance stations and hospital locations in the Central Norway region, 2022. Background image reprinted from www.geodata.no (Esri, Kartverket, Geovekst, Kommuner, OSM, USGS, Garmin, FAO, NOAA) under a CC BY 4.0 license, with permission from Geodata AS, original copyright 2023

EMCC Sør-Trøndelag covers a mixed rural/urban catchment area of 17,830 square kilometers in Sør-Trøndelag, providing healthcare services to approximately 43,000 children under twelve years of age as of November 2022 (Fig. 1) [23]. This area is covered by 22 ambulance bases and one HEMS base.

### Selection

From January 1, 2018, to December 31, 2022, we included all emergency calls to EMCC Sør-Trøndelag involving children under the age of twelve that led to a dispatch of an ambulance and/or helicopter. We defined this age cutoff because anatomy and physiology beyond puberty more closely resembles adult conditions [24]. Neonates, defined as infants aged 0–28 days, were also included. Primary transports, i.e. patients first encountered outside a hospital, were included. Secondary transports, i.e. inter-hospital transports, were not included. We excluded incidents with missing data or insufficient documentation of variables investigated in this study.

### Data source and variables

We extracted data such as patient age, sex, primary medical problem, severity, and transport destination from the EMCC record system AMIS (CSAM Health AS, Oslo, Norway). Additional data for the HEMS patients such as dispatch mode (i.e. helicopter or rapid response car), diagnosis categories, medical interventions and National Advisory Committee for Aeronautics (NACA) severity score, were extracted from the HEMS record system LABAS (Normann IT, Trondheim, Norway). The NACA score is an eight-level scoring system widely recognized for assessing patient severity during the pre-hospital phase, and is assigned to every patient by the HEMS physician [25]. The NACA score has demonstrated its effectiveness in predicting mortality and is also a suitable scoring system for children [26, 27]. Moreover, a diagnosis based on the ICD-10 classification (International Classification of Disease, Tenth Revision, ICD-10) is made by the HEMS physician after each mission [25, 28].

### Statistical analysis

Descriptive statistics are presented as median and interquartile range (IQR) for continuous data. Categorical data are presented as counts and percentages. Incidence rates were reported as the number of events per 1000 person-years in the relevant population, with 95% confidence intervals, and calculated by Poisson regression [29]. All the statistical analyses were performed using SPSS® Statistics (version 29.0.2.0; IBM Corp., Armonk, NY, USA).

## Results

**Fig. 2:**
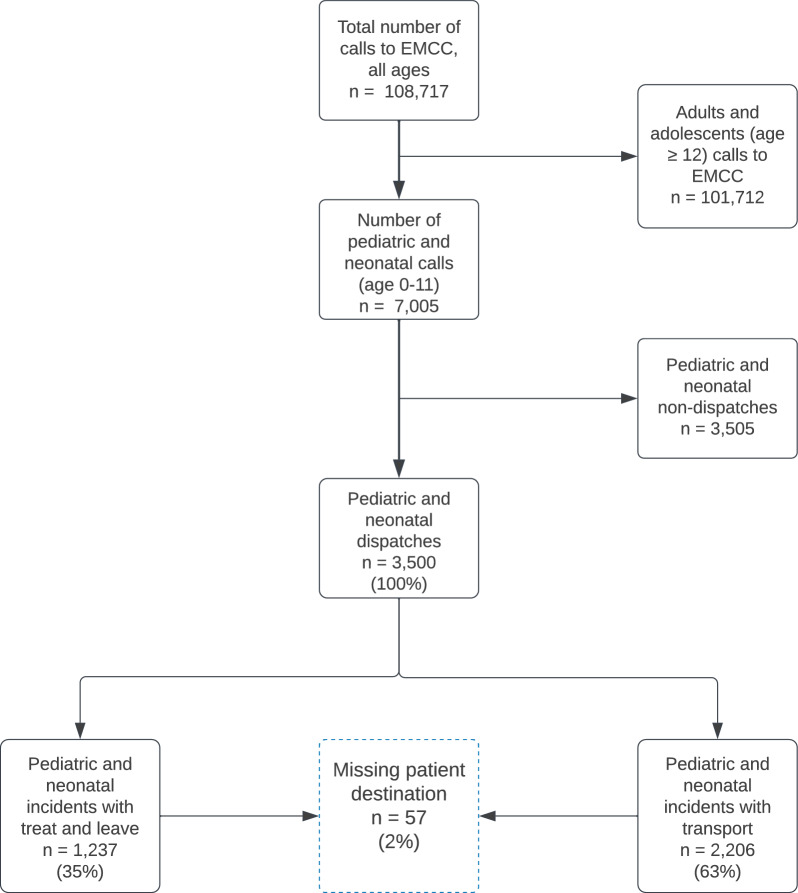
Flowchart of emergency calls to EMCC Sør-Trøndelag, and the inclusion of patients aged 0–11 years during the study period 2018–2022

During the five-year study period, there were 108,717 emergency calls to the EMCC, of which 7005 (6%) concerned children under twelve years of age. Of these, 3500 (3%) lead to a dispatch (Fig. 2). This gives an annual incidence of dispatches of 17 per 1000 children in the population of 43,000. A total of 1560 (45%) incidents resulted in transport to a hospital and 646 (18%) to a general practitioner (Fig. 2). Out of the pediatric and neonatal dispatches, 91% were unique ambulance dispatches and 0.2% were unique HEMS dispatches. In 8% of the cases, both ambulance and HEMS were dispatched. All patients handled by HEMS alone were transported.

Of the HEMS dispatches, 40% were helicopter dispatches, and 60% were rapid-response car dispatches. The four municipalities closest to the HEMS base received 166 (92%) of all the rapid response car dispatches. In the incidents where both ambulance and HEMS resources were involved, 45% of the patients were transported to a healthcare facility by ambulance alone, while the HEMS physician accompanied the ambulance in 19% of the transports. In 29% of these cooperative incidents, the transport was by helicopter.

There was a total of 3551 patients and 3500 dispatches (51 more patients than transports); in most cases this was due to traffic accidents with multiple patients and EMS resources involved.

**Fig. 3:**
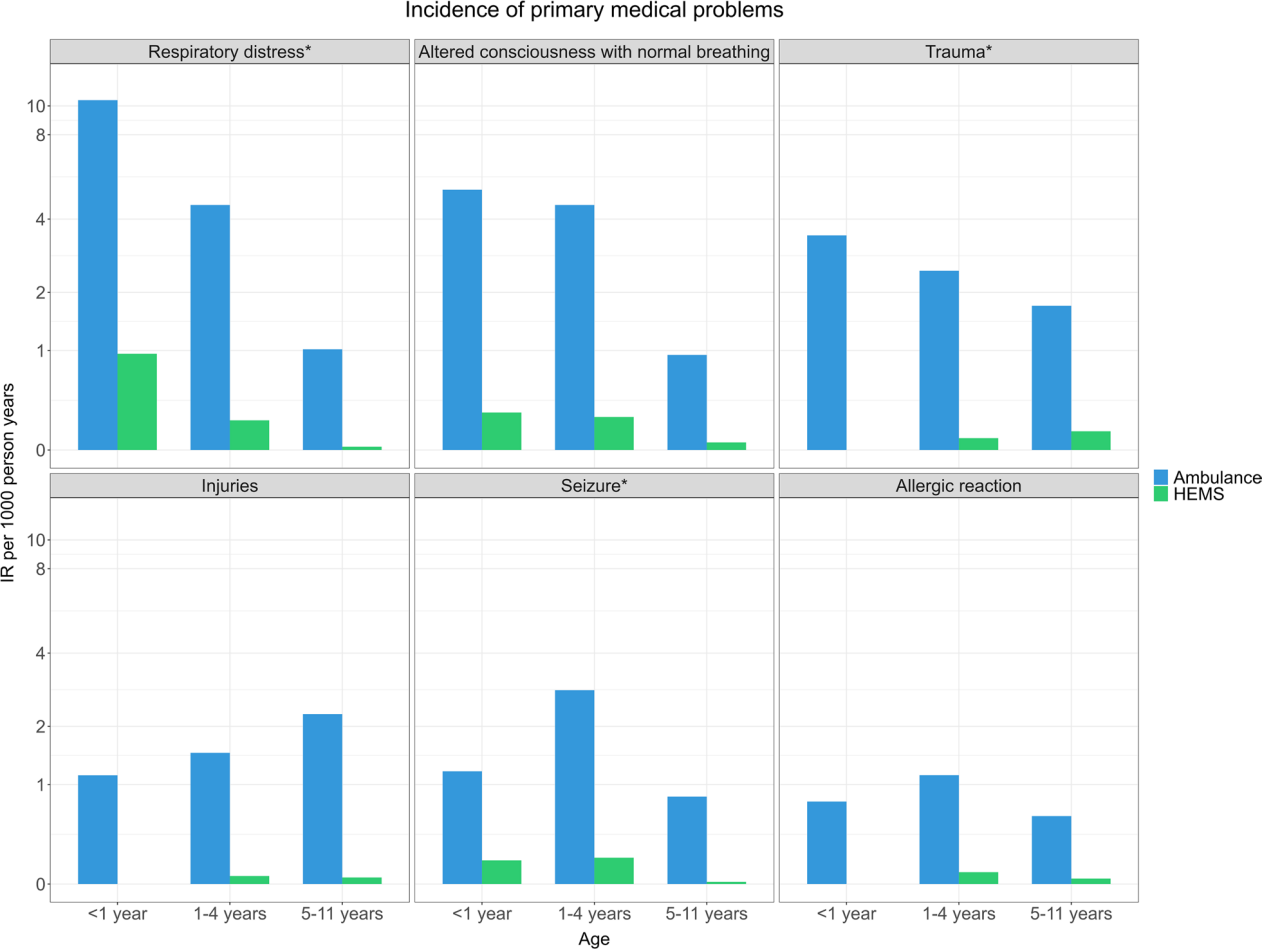
Incidence rate per 1000 person-years of the top six primary medical problems in different age groups in unique ambulance and HEMS incidents. Highlighted assessments (*) reflect those conditions that explicitly align with published PECARN priorities for pediatric pre-hospital research [4]

The “primary medical problem” determined by the EMCC operator at the time of the emergency call is listed in Table 1. Regarding unique ambulance patients, 17% suffered from respiratory distress, where the top three subgroups were “breathing difficulties”, “barely able to breathe”, and “not able to speak coherently due to respiratory difficulties”. The incidence of primary medical problems is shown in Fig. 3. The figure indicates that the younger the patient, the more frequently HEMS is involved in the care of children with respiratory distress and altered consciousness. In contrast, for patients suffering from trauma, HEMS involvement increases with the patient’s age.

**Table 1:** Demographics and clinical characteristics of patients (n = 3 551) treated by EMS Sør-Trøndelag 2018—2022

	n (%)			
	Total n = 3 551	Unique ambulance patients n = 3 242 (91)	Unique HEMS patients n = 8 (0,2)	Ambulance and HEMS patients n = 301 (8)
*Sex*				
Boys	2 006 (56)	1 822 (56)	4 (50)	180 (60)
Unknown sex	89 (3)	89 (3)	0	0 (0)
*Age, median (IQR)*	3 (6)	3 (6)	4 (7)	2 (1)
< 1 years infants	570 (16)	507 (16)	0 (0)	63 (21)
1—4 years preschool	1 699 (48)	1 536 (47)	4 (50)	159 (53)
5—11 years school-age	1 282 (36)	1 199 (37)	4 (50)	79 (26)
*Primary Medical Problem*				
Respiratory distress*	623 (18)	577 (17)	1 (12)	45 (15)
Altered level of consciousness with normal breathing	530 (15)	482 (15)	0 (0)	48 (16)
Trauma*	467 (13)	437 (13)	2 (25)	28 (9)
Other	427 (12)	371 (11)	2 (25)	54 (18)
Injuries	422 (12)	408 (13)	2 (25)	12 (4)
Seizure*	332 (9)	298 (9)	0 (0)	34 (11)
Allergic reaction	173 (5)	155 (5)	0 (0)	18 (6)
Fever/infections	117 (3)	112 (3)	0 (0)	5 (2)
Airway obstruction with foreign body	96 (3)	80 (2)	0 (0)	16 (5)
Altered level of consciousness without normal breathing	95 (3)	72 (2)	0 (0)	23 (8)
*Pre-hospital Severity*				
Acute	2 675 (75)	2 367 (73)	7 (88)	301 (100)
Urgent	871 (25)	870 (27)	1 (12)	0 (0)
Ordinary	5 (0)	5 (0)	0 (0)	0 (0)

Highlighted assessments (*) reflect those conditions that explicitly align with published PECARN priorities for pediatric pre-hospital research [4]

The ICD-10 diagnosis made by the HEMS physician is presumably more accurate than the primary medical problem set by the EMCC operator, because the HEMS physician has examined the patient on scene, and more information is normally available later in the pre-hospital phase. In Table 2 we have categorized similar ICD-10 diagnoses into groups of primary diagnoses. The top three diagnosis groups set by the HEMS physician were seizure, trauma, and respiratory distress. Regarding pre-hospital interventions, 131 (42%) of the HEMS patients received one or more advanced medical interventions. The median NACA score for HEMS patients was 3 (1 = 2, Q2 = 3, Q3 = 3). Detailed clinical characteristics of the HEMS patients are found in Table 2.

**Table 2:** Unique clinical characteristics of patients (n = 309) treated and transported by HEMS Sør-Trøndelag 2018—2022

*Primary diagnosis based on ICD-10*	n (%) or median (IQR)
	Total n = 309
Seizure*	82 (27)
R56.0 Febrile convulsions	42
R56 Convulsions, not elsewhere classified	14
G40 Epilepsy	13
G41Status epilepticus	6
R56.8 Other and unspecified convulsions	5
Trauma*	41 (13)
S72 Fracture of femur	5
S01.0 Open wound of scalp	5
S06.1 Traumatic cerebral oedema	3
S01.5 Open wound of lip and oral cavity	2
S09.9 Unspecified injury of head	2
S36 Injury of intra-abdominal organs	2
T75.1 Drowning and nonfatal submersion	2
Respiratory distress*	38 (12)
J96.0 Acute respiratory failure	12
J05.0 Acute obstructive laryngitis	6
P22 Respiratory distress of newborn	4
J04.0 Acute laryngitis	3
J96 Respiratory failure, not elsewhere classified	3
J04 Acute laryngitis and tracheitis	2
J45 Asthma	2
Injuries	30 (10)
S06.0 Concussion	20
S20 Superficial injury of thorax	2
S30 Superficial injury of abdomen, lower back and pelvis	2
Allergic reaction	20 (6)
T78.4 Allergy, unspecified	11
T78.1 Other adverse food reactions	5
T78.2 Anaphylactic shock, unspecified	2
Gastrointestinal/abdominal	19 (6)
T18 Foreign body in alimentary tract	5
R10 Abdominal and pelvic pain	3
T18.1 Foreign body in oesophagus	3
R10.0 Acute abdomen	2
R10.1 Pain localized to upper abdomen	2
Fever/infections	16 (5)
J22 Unspecified acute lower respiratory infection	5
J06.9 Acute upper respiratory infection	3
B99 Other and unspecified infectious diseases	2
J06 Acute upper respiratory infections of multiple and unspecified sites	2
Airway obstruction with foreign body	12 (4)
T17 Foreign body in respiratory tract	9
Cardiac arrest*	7
I46 Cardiac arrest	7
Altered level of consciousness with normal breathing	5 (2)
R55 Syncope and collapse	4
Patients requiring interventions(s)**	131 (42)
Assisted ventilation*	28 (9)
Resuscitation	14 (5)
Intubation *	10 (3)
Interosseous vascular access	4 (1)
Blood transfusion	2 (2)
Defibrillation	1 (0)
*NACA score*	
0	0 (0)
1	7 (2)
2	30 (10)
3	162 (52)
4	75 (24)
5	28 (9)
6	6 (2)
7	5 (2)

ICD-10: International Classification of Disease, version 10 [28]

*Highlighted assessments reflect those clinical topics that explicitly align with published PECARN priorities for pediatric pre-hospital research [4]

**A unique patient may receive multiple interventions. Percentages indicate interventions performed divided by all patients treated by a HEMS physician

A total of eight patients in our population died within 24 h of emergency transport, six boys and two girls. No patients died within the next 29 days of transport, thus yielding a cumulative 30-day prevalence of 2‰. In five of the eight deaths, HEMS were involved, and three were unique ambulance patients. In the incidents involving HEMS, four patients suffered from cardiac arrest (ICD-code I46), and one from sudden infant death syndrome (ICD-10 code R95). The primary medical problems of the three unique ambulance patients were trauma, respiratory distress, and reduced consciousness.

## Discussion

We found that 6% of EMCC calls in our region involved children under twelve years of age, and 50% of these led to a dispatch of an EMS unit. Given a population of approximately 43,000, there was an annual incidence of dispatches of 17 per 1000 children. Of these, 91% were handled by ambulance alone, 8% were handled by ambulance supported by HEMS, and under 1% were handled by HEMS alone. The most common primary medical problems for children receiving pre-hospital care in our region were respiratory distress, altered consciousness, and trauma. The most common medical interventions provided by HEMS physicians were assisted ventilation, resuscitation, and endotracheal intubation.

The prevalence of pediatric EMS patients in our study aligns well with those reported by Dryana et al. and Richard et al., where the prevalence was 8% and 5%, respectively [2, 14]. Further, we found that 50% of the emergency calls regarding children did not lead to an EMS dispatch. There may be different reasons for this. Firstly, the emergency call may have turned out not to represent an emergency after all. Secondly, medical advice from the EMCC operator could have reassured and facilitated the caller to solve minor medical problems over the phone. Finally, the EMCC operator could have interpreted the patient not to be in need of an EMS response, but rather to be able to present at the appropriate healthcare facility with his/her family. The latter is an essential function of the EMCC operators, as they are the role of a gatekeeper to the limited pre-hospital EMS resources [30]. We are not able to study the emergency calls that did not lead to an EMS dispatch, but further studies should examine the characteristics of these calls to ensure that these children are not under-triaged.

In our study, 35% of the patients were assessed and treated by EMS—but not transported. Several studies from various EMSs have found that this patient group varies extensively from 12 to 44% [1, 2, 31]. There might be different reasons for not transporting a patient. It could be that the medical problem has resolved itself from the time of the emergency call to the arrival of HEMS, e.g., seizures [2]. In some instances, the EMS staff probably recommended the parents or caregivers to bring the child to a healthcare facility themselves (e.g., a general practitioner) for a check-up. It is important to note that the decision not to transport the patient by ambulance only refers to the means of transport, not the need for medical care.

While the EMCC classified all incidents assigned to HEMS as acute, the mean NACA score for these patients where 3, which is classified as severe but not life-threatening [25]. Similar findings were reported by Larsson et. al. and Khorram-Manesh et. al [32, 33]. The moderate NACA score could reflect that the barrier for dispatching HEMS to children is low. An American study by Knofsky et. al. found that pediatric patients transported by HEMS are less severely injured compared to adult patients based on lower Injury Severity Score [34].

Primary medical problems, set by the EMCC operator at the time of emergency call, are mainly used to assign each emergency call a severity grade to help decide with which severity to alert ambulance and HEMS. In this study, we have used these medical problems to describe the pre-hospital pediatric population. However, primary medical problems marked as “Others” account for a substantial number of these problems and make it challenging to describe the pre-hospital pediatric EMS population as a whole. The most common primary medical problems for the children in our study were respiratory distress, altered consciousness, and trauma. Similar findings were reported by Drayna et al. in an American study from 2015, with the top three primary problems being respiratory distress, seizure, and blunt trauma [14, 31]. In our study, medical conditions were more common than trauma and injuries, which differs from the findings of other studies where injuries dominated [31, 35]. A possible explanation for this difference could be that we did not include children older than 11 years, representing the majority age group of the injury category in these studies. Another explanation could be that Norway has separate emergency departments and out-of-hours primary healthcare services, so children with minor injuries may be referred to an out-of-hours primary healthcare service without the involvement of EMS [18].

Our study identified six of the fifteen clinical high-priority topics as defined by PECARN; four primary medical problems (seizure, trauma, respiratory distress and cardiac arrest) and two pre-hospital interventions; (assisted ventilation and intubation) [4]. Understanding how these clinical topics are represented in the pediatric population can provide valuable information for further research. The most common ICD-10 diagnosis among HEMS patients in our study was seizures, accounting for 27% of cases. Similarly, Enomoto et al. identified seizures as the most common non-traumatic incident type. However, only 9% of their population experienced seizures [36]. We found that 2% of the HEMS population experienced cardiac arrest. Similar results were reported in a German study by Mockler et al., which found that 3% of the pediatric population suffered from cardiac arrest [37].

Pre-hospital advanced medical interventions were provided to 131 (42%) of the HEMS patients. This is more common than the findings reported by Nielsen et al., who reported that 20% of all pediatric patients in Danish HEMS received advanced medical interventions [19]. In our study, 3% of patients received endotracheal intubation. This is consistent with findings reported by Selig et al. in an Austrian study that reported that 4% received endotracheal intubation, but less common than that reported in a German study by Eich et al., where 8% of the pediatric patients received endotracheal intubation by a HEMS physician [38, 39]. The added competence of a HEMS physician enables more advanced interventions compared to an ambulance alone.

As mentioned, several studies have shown that ambulance staff report heightened anxiety when working with pediatric patients [5, 10–12, 37, 40]. These studies have shown that anxiety increased errors in medication, basic airway management and appropriate administration of oxygen among experienced paramedics [10–12]. Considerable experience with pre-hospital medical problems and interventions regarding adults is not directly transferable to pediatric patients [41]. Combining knowledge of the skills reported as challenging by Hansen et al. and the incidence of pre-hospital medical problems and interventions found in our study, may help define which topics to address in education and training. A way to apply these findings in an educational program could be to use simulation to practice the skills listed in Table 2 in the context of common clinical scenarios as listed in Tables 1 and 2. Ensuring regular training and opportunities to maintain necessary clinical skills for pre-hospital care is documented to reduce anxiety [13].

Our findings confirm that deaths in pre-hospital care are low. Of the eight pre-hospital deaths in our study, seven had a non-traumatic cause. Similar findings are seen in a Danish study by Nielsen et al., where nontraumatic illness accounted for 19 of 23 deaths [19]. In our study, five patients were considered dead upon arrival of HEMS and, therefore, given a NACA score of seven. Identifying and describing patients who died during or shortly after the pre-hospital care, may help identify areas of improvement in pre-hospital care.

### Strengths and limitations

In this study, we utilized data generated by EMCC and EMS staff during the management of emergency incidents. The data were not originally intended to address our specific research questions. Additionally, we had no access to patient data documented by the ambulance staff. The analysis is limited to patient data documented by EMCC operators and HEMS physicians. The EMCC operator’s documentation is primarily based on secondhand information from the caller and, therefore, will be associated with a lower level of certainty. The role of the EMCC operator is, first and foremost, to identify potentially life-threatening conditions and to prioritize limited EMS resources. Consequently, there is a substantial amount of nonspecific and incomplete data regarding primary medical problems labeled “others”. This makes it challenging to characterize the population fully. The patient documentation by HEMS physicians is first-hand documentation with a higher level of certainty. However, data from the HEMS record system LABAS originates from a few patients, making it difficult to draw any conclusions.

Finally, a significant proportion of pediatric emergency calls did not lead to a dispatch of either ambulance or HEMS. Little is known about these patients, and further research is needed to describe this population.

## Conclusion

Pediatric patients comprise a small part of the population in pre-hospital emergency medical services in our region. We identified respiratory distress, reduced consciousness, and trauma as the most common primary medical problems for children receiving pre-hospital care by EMS in Sør-Trøndelag. Moreover, we identified assisted ventilation and resuscitation as the most frequent advanced medical interventions provided by HEMS physicians. The low frequency of pediatric EMS transports and medical interventions confirms earlier findings, that EMS staff may have insufficient experience in pediatric pre-hospital care. The findings of this study may facilitate the planning of pediatric pre-hospital education and research.

## Supplementary Information

Additional file1.

## Data Availability

Raw data regarding the dataset is not publicly available to protect individuals' privacy, but access may be granted upon request to Helse-Midt regional health authorities.

## Abbreviations

AMIS: Akuttmedisinsk informasjonssystem (a database for emergency calls and EMS response)
CPR: Cardiopulmonary resuscitation
EMCC: Emergency medical communication center
EMS: Emergency medical service
HEMS: Helicopter emergency medical service
NACA: National advisory committee for aeronautics
PECARN: Pediatric emergency care applied research network

## References

[1] Harve H, Salmi H, Rahiala E, Pohjalainen P, Kuisma M. Out-of-hospital paediatric emergencies: a prospective, population-based study. Acta Anaesthesiol Scand. 2016;60(3):360–9. 10.1111/aas.12648. 26489697 10.1111/aas.12648

[2] Richard J, Osmond MH, Nesbitt L, Stiell IG. Management and outcomes of pediatric patients transported by emergency medical services in a Canadian prehospital system. CJEM. 2006;8(1):6–12. 10.1017/s1481803500013312. 17175623 10.1017/s1481803500013312

[3] Andersen K, Mikkelsen S, Jørgensen G, Zwisler ST. Paediatric medical emergency calls to a danish emergency medical dispatch centre: a retrospective, observational study. Scand J Trauma Resusc Emerg Med. 2018;26(1):2. 10.1186/s13049-017-0470-1. 29304841 10.1186/s13049-017-0470-1 PMC5756442

[4] Foltin GL, Dayan P, Tunik M, Marr M, Leonard J, Brown K, et al. Priorities for pediatric prehospital research. Pediatr Emerg Care. 2010;26(10):773–7. 10.1097/PEC.0b013e3181fc4088. 20930604 10.1097/PEC.0b013e3181fc4088

[5] Hansen M, Meckler G, Dickinson C, Dickenson K, Jui J, Lambert W, et al. Children’s safety initiative: a national assessment of pediatric educational needs among emergency medical services providers. Prehosp Emerg Care. 2015;19(2):287–91. 10.3109/10903127.2014.959223. 25296191 10.3109/10903127.2014.959223 PMC4380579

[6] Guise JM, Meckler G, O’Brien K, Curry M, Engle P, Dickinson C, et al. Patient safety perceptions in pediatric out-of-hospital emergency care: children’s safety initiative. J Pediatr. 2015;167(5):1143-8.e1. 10.1016/j.jpeds.2015.07.023. 26297483 10.1016/j.jpeds.2015.07.023 PMC4661065

[7] Cushman JT, Fairbanks RJ, O’Gara KG, Crittenden CN, Pennington EC, Wilson MA, et al. Ambulance personnel perceptions of near misses and adverse events in pediatric patients. Prehosp Emerg Care. 2010;14(4):477–84. 10.3109/10903127.2010.497901. 20662679 10.3109/10903127.2010.497901 PMC2932803

[8] Guise JM, Hansen M, O’Brien K, Dickinson C, Meckler G, Engle P, et al. Emergency medical services responders’ perceptions of the effect of stress and anxiety on patient safety in the out-of-hospital emergency care of children: a qualitative study. BMJ Open. 2017;7(2):e014057. 10.1136/bmjopen-2016-014057. 28246139 10.1136/bmjopen-2016-014057 PMC5337745

[9] Meckler G, Hansen M, Lambert W, O’Brien K, Dickinson C, Dickinson K, et al. Out-of-hospital pediatric patient safety events: results of the CSI chart review. Prehosp Emerg Care. 2018;22(3):290–9. 10.1080/10903127.2017.1371261. 29023218 10.1080/10903127.2017.1371261 PMC6170199

[10] LeBlanc VR, MacDonald RD, McArthur B, King K, Lepine T. Paramedic performance in calculating drug dosages following stressful scenarios in a human patient simulator. Prehosp Emerg Care. 2005;9(4):439–44. 10.1080/10903120500255255. 16263679 10.1080/10903120500255255

[11] Lammers RL, Willoughby-Byrwa M, Fales WD. Errors and error-producing conditions during a simulated, prehospital, pediatric cardiopulmonary arrest. Simul Healthc. 2014;9(3):174–83. 10.1097/sih.0000000000000013. 24401924 10.1097/SIH.0000000000000013

[12] Lammers RL, Byrwa MJ, Fales WD, Hale RA. Simulation-based assessment of paramedic pediatric resuscitation skills. Prehosp Emerg Care. 2009;13(3):345–56. 10.1080/10903120802706161. 19499472 10.1080/10903120802706161

[13] Stevens SL, Alexander JL. The impact of training and experience on EMS providers’ feelings toward pediatric emergencies in a rural state. Pediatr Emerg Care. 2005;21(1):12–7. 10.1097/01.pec.0000150982.96357.ca. 15643317 10.1097/01.pec.0000150982.96357.ca

[14] Drayna PC, Browne LR, Guse CE, Brousseau DC, Lerner EB. Prehospital pediatric care: opportunities for training, treatment, and research. Prehosp Emerg Care. 2015;19(3):441–7. 10.3109/10903127.2014.995850. 25658967 10.3109/10903127.2014.995850

[15] Lerner EB, Dayan PS, Brown K, Fuchs S, Leonard J, Borgialli D, et al. Characteristics of the pediatric patients treated by the pediatric emergency care applied research network’s affiliated EMS agencies. Prehosp Emerg Care. 2014;18(1):52–9. 10.3109/10903127.2013.836262. 24134593 10.3109/10903127.2013.836262

[16] Shah MN, Cushman JT, Davis CO, Bazarian JJ, Auinger P, Friedman B. The epidemiology of emergency medical services use by children: an analysis of the national hospital ambulatory medical care survey. Prehosp Emerg Care. 2008;12(3):269–76. 10.1080/10903120802100167. 18584491 10.1080/10903120802100167 PMC5237581

[17] von Elm E, Altman DG, Egger M, Pocock SJ, Gøtzsche PC, Vandenbroucke JP. Strengthening the reporting of observational studies in epidemiology (STROBE) statement: guidelines for reporting observational studies. BMJ. 2007;335(7624):806–8. 10.1136/bmj.39335.541782.AD. 17947786 10.1136/bmj.39335.541782.AD PMC2034723

[18] Nieber T, Hansen EH, Bondevik GT, Hunskår S, Blinkenberg J, Thesen J, et al. Organization of Norwegian out-of-hours primary health care services. Tidsskr Nor Laegeforen. 2007;127(10):1335–8. 17519984

[19] Nielsen VML, Bruun NH, Søvsø MB, Kløjgård TA, Lossius HM, Bender L, et al. Pediatric emergencies in helicopter emergency medical services: a national population-based cohort study from Denmark. Annals Emerg Med. 2022;80(2):143–53. 10.1016/j.annemergmed.2022.03.024. 10.1016/j.annemergmed.2022.03.024 35527122

[20] Hardeland C, Dreyer K, Hesselberg N, Einvik S, Eielsen O, Hansen A, et al. Norsk indeks for medisinsk nødhjelp 4. utgave 2018 english version [Internet]. nakos.no: nakos; 2018 [cited 2023 31. March]. Available from: https://www.nakos.no/pluginfile.php/1269/block_html/content/2019%20engelske%20hjelpetekster%20NIMN%204%20nav.pdf

[21] Ulvin OE, Skjærseth EÅ, Haugland H, Thorsen K, Nordseth T, Orre MF, et al. The introduction of a regional Norwegian HEMS coordinator: an assessment of the effects on response times, geographical service areas and severity scores. BMC Health Serv Res. 2022;22(1):1020. 10.1186/s12913-022-08337-z. 35948977 10.1186/s12913-022-08337-z PMC9365225

[22] Nakstad AR, Sørebø H, Heimdal HJ, Strand T, Sandberg M. Rapid response car as a supplement to the helicopter in a physician-based HEMS system. Acta Anaesthesiol Scand. 2004;48(5):588–91. 10.1111/j.0001-5172.2004.00395.x. 15101853 10.1111/j.0001-5172.2004.00395.x

[23] 04231: Live births, by region, contents and year [Internet]. Statistisk sentralbyrå. 2023 [cited 31.03.23]. Available from: https://www.ssb.no/en/statbank/table/04231/tableViewLayout1/

[24] Fleming S, Thompson M, Stevens R, Heneghan C, Plüddemann A, Maconochie I, et al. Normal ranges of heart rate and respiratory rate in children from birth to 18 years of age: a systematic review of observational studies. Lancet. 2011;377(9770):1011–8. 10.1016/S0140-6736(10)62226-X. 21411136 10.1016/S0140-6736(10)62226-X PMC3789232

[25] Raatiniemi L, Mikkelsen K, Fredriksen K, Wisborg T. Do pre-hospital anaesthesiologists reliably predict mortality using the NACA severity score? A retrospective cohort study. Acta Anaesthesiol Scand. 2013;57(10):1253–9. 10.1111/aas.12208. 24134443 10.1111/aas.12208 PMC4287201

[26] Bonatti J, Göschl O, Larcher P, Wödlinger R, Flora G. Predictors of short-term survival after helicopter rescue. Resuscitation. 1995;30(2):133–40. 10.1016/0300-9572(95)00883-u. 8560102 10.1016/0300-9572(95)00883-u

[27] Wendling-Keim DS, Hefele A, Muensterer O, Lehner M. Trauma scores and their prognostic value for the outcome following pediatric polytrauma. Front Pediatr. 2021;9:721585. 10.3389/fped.2021.721585. 34540770 10.3389/fped.2021.721585 PMC8446435

[28] ICD-10 Version:2019 [Internet]. World Health Organization. 2024. Available from: https://icd.who.int/browse10/2019/en

[29] Zou G. A modified Poisson regression approach to prospective studies with binary data. Am J Epidemiol. 2004;159(7):702–6. 10.1093/aje/kwh090. 15033648 10.1093/aje/kwh090

[30] Roivainen P, Hoikka MJ, Raatiniemi L, Silfvast T, Ala-Kokko T, Kääriäinen M. Telephone triage performed by nurses reduces non-urgent ambulance missions: a prospective observational pilot study in Finland. Acta Anaesthesiol Scand. 2020;64(4):556–63. 10.1111/aas.13542. 31898315 10.1111/aas.13542

[31] Joyce SM, Brown DE, Nelson EA. Epidemiology of pediatric EMS practice: a multistate analysis. Prehosp Disaster Med. 1996;11(3):180–7. 10.1017/s1049023x00042928. 10163380 10.1017/s1049023x00042928

[32] Larsson G, Larsson S, Strand V, Magnusson C, Andersson HM. Pediatric trauma patients in Swedish ambulance services -a retrospective observational study of assessments, interventions, and clinical outcomes. Scand J Trauma Resusc Emerg Med. 2024;32(1):51. 10.1186/s13049-024-01222-0. 38840226 10.1186/s13049-024-01222-0 PMC11151517

[33] Khorram-Manesh A, Lennquist Montán K, Hedelin A, Kihlgren M, Örtenwall P. Prehospital triage, discrepancy in priority-setting between emergency medical dispatch centre and ambulance crews. Eur J Trauma Emerg Surg. 2011;37(1):73–8. 10.1007/s00068-010-0022-0. 26814754 10.1007/s00068-010-0022-0

[34] Knofsky M, Burns JBJ, Chesire D, Tepas JJ, Kerwin AJ. Pediatric trauma patients are more likely to be discharged from the emergency department after arrival by helicopter emergency medical services. J Trauma Acute Care Surg. 2013;74(3):917–20. 10.1097/TA.0b013e31827e19a4. 23425758 10.1097/TA.0b013e31827e19a4

[35] Seidel JS, Henderson DP, Ward P, Wayland BW, Ness B. Pediatric prehospital care in urban and rural areas. Pediatrics. 1991;88(4):681–90. 1896270

[36] Enomoto Y, Tsuchiya A, Tsutsumi Y, Kikuchi H, Ishigami K, Osone J, et al. Characteristics of children cared for by a physician-staffed helicopter emergency medical service. Pediatr Emerg Care. 2021;37(7):365–70. 10.1097/pec.0000000000001608. 30211837 10.1097/PEC.0000000000001608

[37] Mockler S, Metelmann C, Metelmann B, Thies KC. Prevalence and severity of pediatric emergencies in a German helicopter emergency service: implications for training and service configuration. Eur J Pediatr. 2023;182(11):5057–65. 10.1007/s00431-023-05178-8. 37656240 10.1007/s00431-023-05178-8 PMC10640406

[38] Selig HF, Trimmel H, Voelckel WG, Hüpfl M, Trittenwein G, Nagele P. Prehospital pediatric emergencies in Austrian helicopter emergency medical service – a nationwide, population-based cohort study. Wien Klin Wochenschr. 2011;123(17):552–8. 10.1007/s00508-011-0006-z. 21691755 10.1007/s00508-011-0006-z

[39] Eich C, Roessler M, Nemeth M, Russo SG, Heuer JF, Timmermann A. Characteristics and outcome of prehospital paediatric tracheal intubation attended by anaesthesia-trained emergency physicians. Resuscitation. 2009;80(12):1371–7. 10.1016/j.resuscitation.2009.09.004. 19804939 10.1016/j.resuscitation.2009.09.004

[40] Cottrell EK, O’Brien K, Curry M, Meckler GD, Engle PP, Jui J, et al. Understanding safety in prehospital emergency medical services for children. Prehosp Emerg Care. 2014;18(3):350–8. 10.3109/10903127.2013.869640. 24669906 10.3109/10903127.2013.869640 PMC4062591

[41] Gillis J, Loughlan P. Not just small adults: the metaphors of paediatrics. Arch Dis Child. 2007;92(11):946–7. 10.1136/adc.2007.121087. 17954476 10.1136/adc.2007.121087 PMC2083631

