# Supplementary material for: Pre-hospital care for children: a descriptive study from Central Norway
Source: Scand J Trauma Resusc Emerg Med. 2024 Nov 4;32:106. doi: 10.1186/s13049-024-01279-x (PMC11536780; doi:10.1186/s13049-024-01279-x)
Supplement: Supplementary file 1 — Additional file1. [file 13049_2024_1279_MOESM1_ESM.docx]

**Dispatch criteria for Helicopter Emergency Medical Services**
EMCC Sør-Trøndelag
Protocol #16577 (accessed 1.2.2024)

**Dispatch request criteria**HEMS should be requested when:

1. Time to definite treatment requires helicopter transport.
2. Medical condition affecting ABCD-status, and physician competency is required for optimal patient trajectory.
3. Access to severely ill or injured patients can only be obtained by HEMS

**Medical conditions where HEMS dispatch request may be applicable**

- Any condition with severe vital function failure
- Cardiac arrest
- Cerebral ischemic event if time to thrombolytic treatment can be mitigated by > 30 min.
- STEMI if time to PCI can be mitigated by > 30 min.
- Severe trauma if national trauma criteria can be applied by anatomical or physiologic criteria.
- Traumatic amputations in upper- or lower extremities
- Drowning
- Avalanche
- Hypothermia
- Preterm childbirth or obstetric complications

**Secondary transports**

**Background:**

Patients that are subject to inter-hospital transfers have to be coordinated and accepted by both transferring and receiving hospital upon HEMS request. Indication for transfer and time frame must be stated in the request. Upon every request risk/reward ratio should be assessed. STEMI patients and cerebral ischemic incidents subject to thrombectomy with ongoing thromolytic treatment is always eligible for transfer.

**Indications:**

- Major medical benefit by rapid transportation to definite treatment
- Requirement for advanced monitoring or treatment by anaesthesiologist during transport
- Alternative transport involving significant risk for patient

**General guidelines**

On call HEMS physician has final authority to accept or decline dispatch request. This assessment is based on medical condition in question, resource availability and expected benefit for the individual patient.

**Critical conditions with vital function failure**The following is examples of conditions where HEMS utilization should be considered:

**Airways**

- Anaphylaxis with threat to airway.
- Airway obstruction.
- Epiglottitis.
- Burns- or inhalation trauma.
- Infections causing stridor or severe dyspnoea.

**Respiratory**

- Respiratory failure or severe dyspnoea with loss of consciousness, that does not respond to interventions available to basic EMS.
- Paediatric patients with subcostal or jugular retractions, with affected consciousness

**Circulation**

- Hypotension not responding to crystalloid treatment
- Neurogenic shock
- Cardiogenic shock or pulmonary edema
- Anaphylactic shock not responding to epinephrine

**Disability**

- Status epilepticus
- Head injuries with loss of consciousness.
- Loss of consciousness with no known aetiology

**Other situations where HEMS utilization may be eligible**

- Patient inaccessibility to basic HEMS or other transportation.
- Acute need for transportation of specific equipment, blood products or medications.
- Search- and rescue missions
